# Association between medial femoral resection thickness and postoperative pain after fixed-bearing unicompartmental knee arthroplasty: an exploratory cohort study

**DOI:** 10.1186/s12891-026-09694-0

**Published:** 2026-03-06

**Authors:** Hironao Shioiri, Tsuneari Takahashi, Katsushi Takeshita

**Affiliations:** 1 Department of Orthopaedic Surgery, Haga Red Cross Hospital, Moka, Japan; 2 Department of Orthopaedics, Jichi Medical University, Shimotsuke, Japan

**Keywords:** Unicompartmental knee arthroplasty, Postoperative pain, Medial femoral resection, Surgical technique

## Abstract

**Background:**

Unicompartmental knee arthroplasty (UKA) is an established treatment for isolated compartment osteoarthritis and is associated with favorable functional outcomes and implant survivorship. However, determinants of achieving a near pain-free state after fixed-bearing (FB) UKA remain incompletely understood. This exploratory study investigated whether medial femoral resection thickness is associated with postoperative pain following medial FB-UKA.

**Methods:**

This retrospective single-surgeon cohort study included 40 consecutive patients who underwent medial fixed-bearing MOTO-UKA between March 2023 and June 2024. Pain was assessed one year postoperatively using the Numerical Rating Scale (NRS). Patients were categorized as near pain-free (NRS ≤ 1.0) or higher pain (NRS > 1.0). Medial femoral resection thickness was measured intraoperatively using calibrated calipers. Univariate analyses were performed. Receiver operating characteristic (ROC) analysis was conducted to explore discrimination.

**Results:**

At one year, 24 patients (60%) achieved NRS ≤ 1.0. In univariate analysis, greater medial femoral resection thickness was observed in the near pain-free group (6.9 ± 1.1 mm vs. 5.8 ± 0.7 mm; *P* = 0.001). ROC analysis demonstrated moderate discrimination (AUC 0.779; 95% CI 0.642–0.916), with an exploratory reference value of 6.5 mm.

**Conclusions:**

In this exploratory single-surgeon cohort of medial fixed-bearing UKA, smaller medial femoral resection thickness was observed in patients reporting higher pain at one year. Resection thickness likely reflects medial compartment balance rather than representing a direct surgical target. Given the limited sample size, these findings should be considered hypothesis-generating and require prospective multicenter validation.

## Introduction

Unicompartmental knee arthroplasty (UKA) is an established surgical treatment for osteoarthritis confined to a single compartment of the knee. Compared with total knee arthroplasty (TKA), UKA provides effective pain relief and functional restoration while being associated with lower morbidity and mortality rates [1]. Registry data indicate that fixed-bearing (FB) implants account for more than 60% of UKA procedures in Australia and the United Kingdom [2]. Reported revision rates for FB-UKA are approximately 3.6–4.8% at five years and 6.4–8.3% at ten years [3, 4]. In addition, UKA is associated with fewer perioperative complications and shorter hospital stays compared with TKA [5].

Optimal patient selection remains essential for favorable outcomes. Satisfaction rates of up to 91% have been reported [6]; however, criteria defining optimal candidates remain debated. Younger age and mild residual varus alignment have been associated with improved outcomes [7]. In TKA, lower postoperative NRS pain scores have been linked to greater satisfaction [8, 9].

Although the incidence of substantial residual pain after UKA is low [1], determinants of achieving a near pain-free state remain incompletely understood. Few studies have specifically evaluated factors associated with minimal residual pain (e.g., NRS ≤ 1.0) following fixed-bearing UKA. We hypothesized that perioperative clinical and radiographic parameters may be associated with such outcomes. The present study was designed as an exploratory analysis to investigate this hypothesis.

## Methods

### Participants

This retrospective study included 40 consecutive patients who underwent fixed-bearing MOTO-UKA (Medacta, Switzerland) between March 2023 and June 2024. Indications were determined by a senior knee surgeon based on symptoms, physical examination, and radiographic findings using the Kellgren–Lawrence (KL) grading system [10]and the Knee Osteoarthritis Grading Scale [11].

Candidates had isolated medial compartment osteoarthritis (KL grade 2,3,4), preserved lateral and patellofemoral compartments confirmed by standing radiographs and intraoperative assessment, and intact anterior cruciate ligament function. All patients had persistent symptoms despite appropriate conservative treatment and elected to undergo UKA after shared decision-making [12]. Patients with prior knee arthroplasty or ligament reconstruction were excluded.

### Variables collected

Collected variables included age, sex, operative side, height, weight, body mass index (BMI), KL grade, preoperative and postoperative Numerical Rating Scale (NRS), preoperative and postoperative knee flexion range of motion (ROM), preoperative hip knee angle (HKA), pre- and postoperative medial proximal tibial angle (MPTA), pre- and postoperative posterior tibial slope (PTS), and bone resection thickness (medial distal femoral condyle, posterior femoral condyle, medial tibial plateau). Bone resection thickness was recorded for the distal medial femoral cut and posterior femoral cut, measured at the thickest point of each resected surface.

### Measurement of medial femoral resection thickness

Medial femoral resection thickness was measured intraoperatively using calibrated surgical calipers at the distal medial femoral cut surface prior to implant placement.

Measurements were taken from the resected bony surface, excluding cartilage wear. Sawblade kerf was not included in the recorded thickness. Thickness was not referenced to the planned implant thickness. All measurements were recorded immediately in a standardized operative record. Inter- and intra-observer reliability were not formally assessed and are acknowledged as limitations.

### Outcome assessment

Pain was assessed one year postoperatively using the NRS (0–10) during in-person outpatient visits. Patients reported average walking pain over the preceding week.

An NRS ≤ 1.0 was selected to represent a stringent “near pain-free” outcome reflecting an optimal postoperative state rather than a merely acceptable one. This threshold does not correspond to established patient-acceptable symptom state (PASS) criteria and should be interpreted as exploratory.

Patients were categorized as: Group G: NRS ≤ 1.0, Group NG: NRS > 1.0.

The term “higher pain at one year” is used to describe Group NG.

### Surgical technique

All procedures were performed by a single experienced knee surgeon using the fixed-bearing MOTO-UKA system. Medial UKA was conducted through a minimally invasive medial parapatellar approach with the patient in the supine position under pneumatic tourniquet control [13]. The surgical technique followed principles comparable to mechanical alignment. A calipered technique was employed to reproduce the patient’s native MPTA and lateral distal femoral angle. Three femoral bone cuts were performed using a tibia-first gap-balancing technique. Resection thickness was measured intraoperatively at the thickest portion of the distal medial femoral cut surface after completion of the cuts. Measurements were obtained based on the resected bony surface, excluding cartilage wear, and sawblade kerf was not included in the recorded thickness. Calipers were used for all measurements. The implant features a fixed-bearing, round-on-flat geometry designed to replicate the anatomical contour of the medial femoral condyle and tibial plateau. This design lacks insert mobility, potentially increasing sensitivity to subtle imbalance. Tibial bone cuts were performed using an extramedullary alignment guide referencing the center of the ankle joint, particularly in cases with slight varus MPTA. Care was taken to avoid medial overstuffing and excessive valgus correction while maintaining appropriate coronal alignment.

### Postoperative rehabilitation

All patients followed a standardized postoperative rehabilitation protocol beginning 3–4 weeks after surgery, consistent with previously reported structured rehabilitation programs after knee arthroplasty [14]. Rehabilitation focused on ROM exercises, quadriceps strengthening, and gait training. Outpatient rehabilitation was supervised and individualized by physical therapists according to patient tolerance and recovery status [9]. Rehabilitation adherence was not formally quantified and is acknowledged as a potential confounding factor.

### Statistical analysis

Continuous variables were expressed as mean ± standard deviation. Categorical variables were presented as counts and percentages. Statistical analyses were conducted using EZR (Saitama Medical Center, Jichi Medical University, Saitama, Japan), a graphical user interface for R [15]. Continuous variables were compared using Student’s t-test or the Mann–Whitney U test where appropriate. Categorical variables were analyzed using Fisher’s exact test.

Given the limited sample size and the small number of patients in the higher-pain group (*n* = 16), multivariable modeling was not performed in order to avoid overfitting and unstable estimates. Therefore, results are presented as univariate comparisons.

Receiver operating characteristic (ROC) analysis was performed to explore the discriminatory performance of medial femoral resection thickness. The area under the curve (AUC) and 95% confidence intervals were calculated using the DeLong method. The optimal reference value was determined using the Youden index. ROC findings are considered exploratory and are not intended for clinical decision-making.

A significance level of *P* < 0.05 was adopted.

## Results

At one year following fixed-bearing UKA, 24 of 40 patients (60%) achieved near pain-free status (NRS ≤ 1.0). All patients demonstrated postoperative knee flexion ≥ 120°. Baseline demographic and clinical characteristics are summarized in Table 1.

**Table 1:** Demographic Data of the Study Population. Values are presented as mean ± standard deviation unless otherwise indicated

Variable	Overall (*n* = 40)
Age (years)	73.8 ± 5.3
Sex (female), n (%)	30 (75.0%)
Height (m)	1.56 ± 0.09
Weight (kg)	59.4 ± 11.8
Body mass index (kg/m²)	24.4 ± 3.9
Kellgren–Lawrence grade, n	Grade 2: 15; Grade 3: 17; Grade 4: 8
Preoperative HKA (° varus)	5.2 ± 4.1
Preoperative MPTA (°)	84.0 ± 12.4
Preoperative tibial slope (°)	9.7 ± 12.5
Preoperative NRS	6.3 ± 2.2

*Abbreviations*: *HKA* hip–knee–ankle angle, *MPTA* medial proximal tibial angle, *ROM* range of motion, *NRS* numerical rating scale

In univariate analysis, no statistically significant differences were observed between groups in age, sex, preoperative HKA, MPTA, PTS, or range of motion. BMI was significantly higher in the higher-pain group (25.9 ± 3.8 vs. 23.4 ± 3.7; *P* = 0.047) (Table 2).

**Table 2:** Comparison between G group and NG Group. Continuous variables are presented as mean ± standard deviation and were compared using Student’s t-test. Categorical variables were analyzed using Fisher’s exact test

Variable	G group (*n* = 24)	NG group (*n* = 16)	*P* value
Age (years)	73.5 ± 6.0	74.4 ± 4.2	0.600
Height (m)	1.55 ± 0.08	1.56 ± 0.10	0.697
Weight (kg)	56.6 ± 10.6	63.5 ± 12.6	0.067
Body mass index (kg/m²)	23.4 ± 3.7	25.9 ± 3.8	0.047
Sex (female), n (%)	18 (75.0%)	12 (75.0%)	1.000
Kellgren–Lawrence grade, n	G2: 10; G3: 9; G4: 5	G2: 5; G3: 8; G4: 3	0.722
Preoperative MPTA (°)	82.5 ± 16.0	86.1 ± 1.8	0.371
Preoperative tibial slope (°)	10.9 ± 15.9	7.9 ± 3.1	0.471
Postoperative MPTA (°)	86.0 ± 3.5	85.1 ± 3.0	0.412
Postoperative tibial slope (°)	6.2 ± 3.1	8.0 ± 3.3	0.082
Preoperative NRS	6.0 ± 2.2	6.7 ± 2.4	0.380
Postoperative 1-year NRS	0.4 ± 0.5	3.4 ± 1.4	< 0.001
Postoperative 1-year ROM (°)	132.0 ± 5.3	129.9 ± 5.9	0.232
Medial femoral resection (mm)	6.9 ± 1.1	5.8 ± 0.7	0.001
Posteromedial femoral resection (mm)	8.4 ± 1.0	8.1 ± 1.4	0.420
Tibial resection (mm)	7.33 ± 1.80	7.31 ± 1.66	0.971

*Abbreviations*: *HKA* hip–knee–ankle angle, *MPTA* medial proximal tibial angle, *ROM* range of motion, *NRS* numerical rating scale

Medial femoral resection thickness was greater in the near pain-free group compared with the higher-pain group (6.9 ± 1.1 mm vs. 5.8 ± 0.7 mm; *P* = 0.001).

**Fig. 1:**
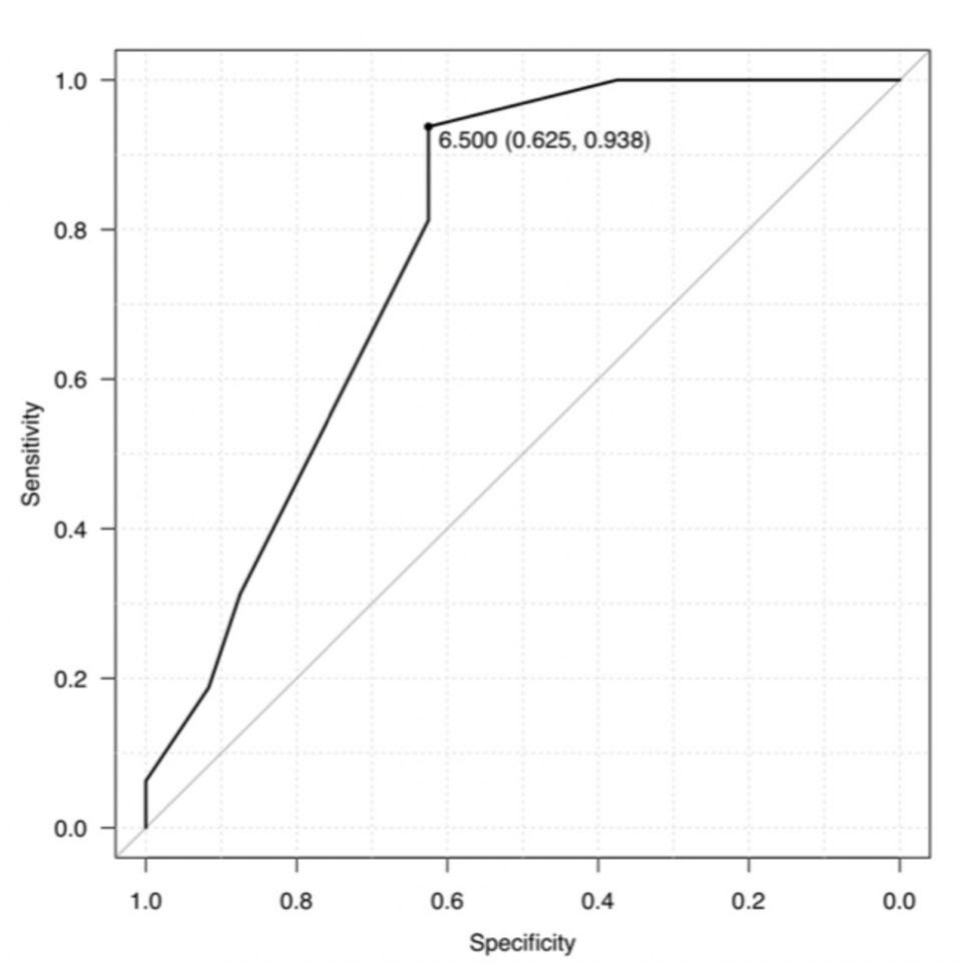
Receiver operating characteristic curve for medial femoral resection thickness predicting higher pain at one year after fixed-bearing unicompartmental knee arthroplasty

ROC analysis demonstrated moderate discrimination (AUC 0.779; 95% CI 0.642–0.916). The optimal exploratory reference value was 6.5 mm, with sensitivity of 62.5% and specificity of 93.8% (Fig. 1). This reference value should be interpreted as hypothesis-generating rather than prescriptive.

## Discussion

The present exploratory study observed an association between smaller medial femoral resection thickness and higher pain at one year following medial fixed-bearing UKA. These findings are based on univariate comparisons within a small cohort and should not be interpreted as evidence of causality.

Medial femoral resection thickness likely functions as a surrogate marker of medial compartment balance rather than an isolated surgical target. In fixed-bearing systems such as the MOTO-UKA, which incorporate a round-on-flat geometry without insert mobility, subtle imbalance may not be accommodated through self-adjustment of the bearing surface. Consequently, relatively small differences in resection thickness may reflect variations in gap tension, ligament strain, and load distribution. Overstuffing of the medial compartment has been recognized as a potential technical pitfall after medial UKA and has been associated with residual medial tightness and altered joint mechanics [16]. Biomechanical investigations have demonstrated that excessive medial compartment filling can increase medial collateral ligament strain and disturb physiological load sharing patterns [17]. Similarly, excessive valgus overcorrection has been linked to unfavorable load redistribution and inferior clinical outcomes [18, 19]. In this context, smaller medial femoral resection thickness may reflect a relatively tighter medial compartment, as resection thickness was determined intraoperatively using a tibia-first gap-balancing technique rather than being predefined according to implant thickness. However, this interpretation remains speculative, does not establish a direct causal relationship, and would require dedicated biomechanical investigation and prospective validation. Postoperative pain after UKA is multifactorial. Factors such as component rotational alignment, ligament quality, psychosocial characteristics, rehabilitation adherence, and pathology in other compartments were not assessed in this study and may have influenced outcomes [20]. BMI may also act as a modifying factor by amplifying load-related imbalance. Therefore, medial femoral resection thickness should be interpreted as reflecting a complex interaction among surgical technique, component positioning, and patient-specific factors.

The stringent NRS ≤ 1.0 threshold used in this study represents an optimal near pain-free state and does not correspond to established PASS definitions. Our objective was to explore determinants of minimal residual pain rather than general satisfaction. Accordingly, the ROC-derived cutoff should not be interpreted as a prescriptive clinical target but rather as a hypothesis-generating reference requiring prospective validation.

Given the limited sample size and the small number of patients in the higher-pain group, the present analysis is statistically fragile and susceptible to instability. The observed association should therefore be interpreted cautiously and confirmed in larger, multicenter cohorts before clinical implications are considered.

### Limitations

Several limitations should be acknowledged when interpreting the findings of this exploratory study.

First, this was a retrospective, single-center study conducted by a single surgeon using a single fixed-bearing implant design, with a relatively small sample size (*n* = 40) and a limited number of events (*n* = 16). These factors substantially limit generalizability. Because multivariable adjustment was not performed in order to avoid overfitting in this small cohort, residual confounding, including the potential influence of BMI and other unmeasured variables, cannot be excluded. Second, all participants were East Asian. Anatomical characteristics, implant sizing considerations, and expectations regarding postoperative outcomes may differ across populations. Therefore, extrapolation to other ethnic or demographic groups should be undertaken cautiously.

Third, several potential confounding variables were not assessed. Psychosocial factors (e.g., anxiety, depression), socioeconomic status, preoperative analgesic use, and rehabilitation adherence were not systematically evaluated, despite their recognized influence on postoperative pain perception. Implant rotational alignment and detailed intraoperative soft-tissue balance measurements were also not analyzed. These unmeasured factors may have influenced the observed association.

Fourth, Formal repeat-measurement testing of resection thickness was not performed, and measurement was obtained at the thickest point of the cut surface, which may introduce variability.Measurement error may therefore have affected the results.

Fifth, follow-up was limited to one year. Longer-term pain trajectories and implant survival outcomes were not assessed. Moreover, the NRS ≤ 1.0 threshold represents an optimal near pain-free state and does not correspond to validated PASS definitions.

Finally, selection bias cannot be excluded. Patients experiencing greater discomfort may have been more likely to attend follow-up evaluations, potentially influencing group distribution.

Given these limitations, the present findings should be interpreted strictly as hypothesis-generating associations rather than causal relationships.

## Conclusion

In this exploratory single-surgeon cohort of medial fixed-bearing UKA, smaller medial femoral resection thickness was statistically associated with higher pain at one year. Resection thickness likely reflects medial compartment balance and implant–soft tissue interaction rather than serving as a direct surgical target. Given the limited sample size and statistical fragility of the model, these findings should be considered preliminary. Prospective, multicenter studies with larger cohorts and detailed biomechanical assessment are required before clinical application can be considered.

## Data Availability

The datasets used and/or analysed during the current study are available from the corresponding author on reasonable request.
